# Bio‐Based Microfluidics With Snail Slime: A By‐Product of Agriculture Plays an Exciting Role in the Chemistry of Microfluidic Reaction Chambers

**DOI:** 10.1002/marc.202500578

**Published:** 2025-10-25

**Authors:** Andrea Koball, Jens Gaitzsch

**Affiliations:** ^1^ Department of Bioactive and Responsive Polymers Leibniz‐Institut Für Polymerforschung Dresden e.V. Dresden Germany

**Keywords:** catalysis, hydrogel, Microfluidics, snail slime, snails

## Abstract

Snail slime, also known as mucus, presents great potential due to its broad spectrum of ingredients, including the eponymous structural proteins (Mucins), glycoproteins, and bioactive compounds such as hyaluronic acid and allantoin. It is most prominently applied in the cosmetic industry as a raw material for the production of protein‐based hybrid hydrogels, and is also known to have potential for synthetic chemistry. For instance, it has been shown to have the ability to form catalytically active gold NPs (Au‐NPs) under mild conditions. In this research, these key features are combined, the ability to reduce gold solutions, stabilize their NPs, and be a chemical building block, for developing Au‐NP‐comprising hydrogel structures from snail slime. Au‐NPs are produced under environmentally friendly conditions and integrated into bio‐based hydrogels for a sustainable reaction process. In the form of micro‐scale dots, the newly designed Au‐NP‐hydrogels are successfully implemented in a microfluidic single‐chamber reactor and utilized for the decolouration (= degradation) of Rhodamine 6G. A path toward a multi‐functional, environmentally friendly microfluidic test chip, utilizing the versatile catalytic activity of green gold NPs, embedded in biogenic and hydrogel materials, is hence presented.

## Graphical abstract

Due to a broad spectrum of active ingredients, snail slime presents a variety of exciting physical properties and chemical reactivities. Especially in the role of the reduction‐ and stabilizing agent for the manufacturing of gold nanoparticles, its great potential is revealed. Utilising the proteins found in slime, Nanoparticle‐comprising hydrogels are produced and adapted for catalytic characterization and applications in microfluidic systems.

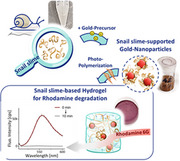

## 1 Introduction

The class of snails, respectively *gastropodae*, currently comprises 721 families divided into about 65 000 species. The order of air‐breathing snails, (*pulmonatae*) with 28 000 known species, draws the common image of snails in society [1]. Although they were originally cultivated as food source [2, 3], snail slime, respectively mucus, has attracted research interest over the recent years due to its’ antibiotic and healing‐promoting properties and the resulting range of possible applications [4, 5, 6]. In particular, studies focus on the identification of active ingredients and characterization of antimicrobial properties [7, 8, 9]. This led to the development of hydrogel‐like anti‐inflammatory, flexible wound dressings, plasters and tissue analogues [10, 11, 12]. Additionally, snail slime is characterized by high water absorbency and consequently with a rehydrating, refreshing effect. Combined with inflammatory and regenerating components, these enabled the manufacturing of skin care products for acne and scar treatment [13, 14]. However, as a typically weakly cross‐linked sol, snail slime presents nearly no spatial stability, thus these promising properties have not been transferred to 3D (hydrogel) structures [15, 16]. Therefore, the potential of the snail slime has only been utilized to a restricted level.

Within the scope of adapting snail slime and its promising properties in synthetic chemistry, Di Fillipo et al. [17] and Gubitosa et al. [18] both presented sustainable procedures for applying snail slime in the synthesis of slime‐supported silver and gold nanoparticles while promoting their antimicrobial and anti‐cancerous qualities [19, 20, 21]. Furthermore, these were characterized by impressive catalytic activity [22, 23, 24]. Exploiting this feature in versatile chemical synthesis required immobilization on suitable porous carrier materials, such as hydrogel networks [25, 26, 27]. For this purpose, a variety of suitable procedures are available, from physical entrapment into developing networks to in‐situ conversion by adding nanoparticle precursor during the formation process [28, 29]. Such structures are in reach by the utilization of the snail slime's own proteins, i.e. biopolymers. Combining snail slime with further biopolymers such as chitosan, cellulose or gelatine enabled the construction of specific spacious, but rigid structures [11, 12] or thin films [30, 31]. Protein‐hybrid‐hydrogels from snail slime‐comprising proteins for 3D, flexible materials in a chemical application would be a logical next step.

Especially the implementation in microfluidic reactors requires suitable immobilization procedures for potential applications in rapid tests, such as blood sugar testing, clinical diagnostic or water quality [32, 33, 34, 35]. The underlying principle of a lab‐on‐chip device relied on feeding minimal sample quantities to a chip, swiftly analyzing for specific parameters and delivering relevant results output in the form of optical signals without dependency on a complete analytical set‐up. Due to the integration of Au‐NPs the indicator reaction, i.e. dye degradation, could be connected to the antimicrobial effect for diagnostic applications [36, 37, 38]. Microfluidic chip systems of this kind currently contain large quantities of mineral oil and gas‐based plastics and are generally designed for single use only, such as common covid‐19 rapid tests [39, 40]. Since our group has recently established a hydrogel‐based microfluidic flow reactor for multiple chemical applications [41, 42, 43, 44, 45], combining the advantages of snail slime as a biogenic resource with microfluidics was rapidly available.

**FIGURE 1:**
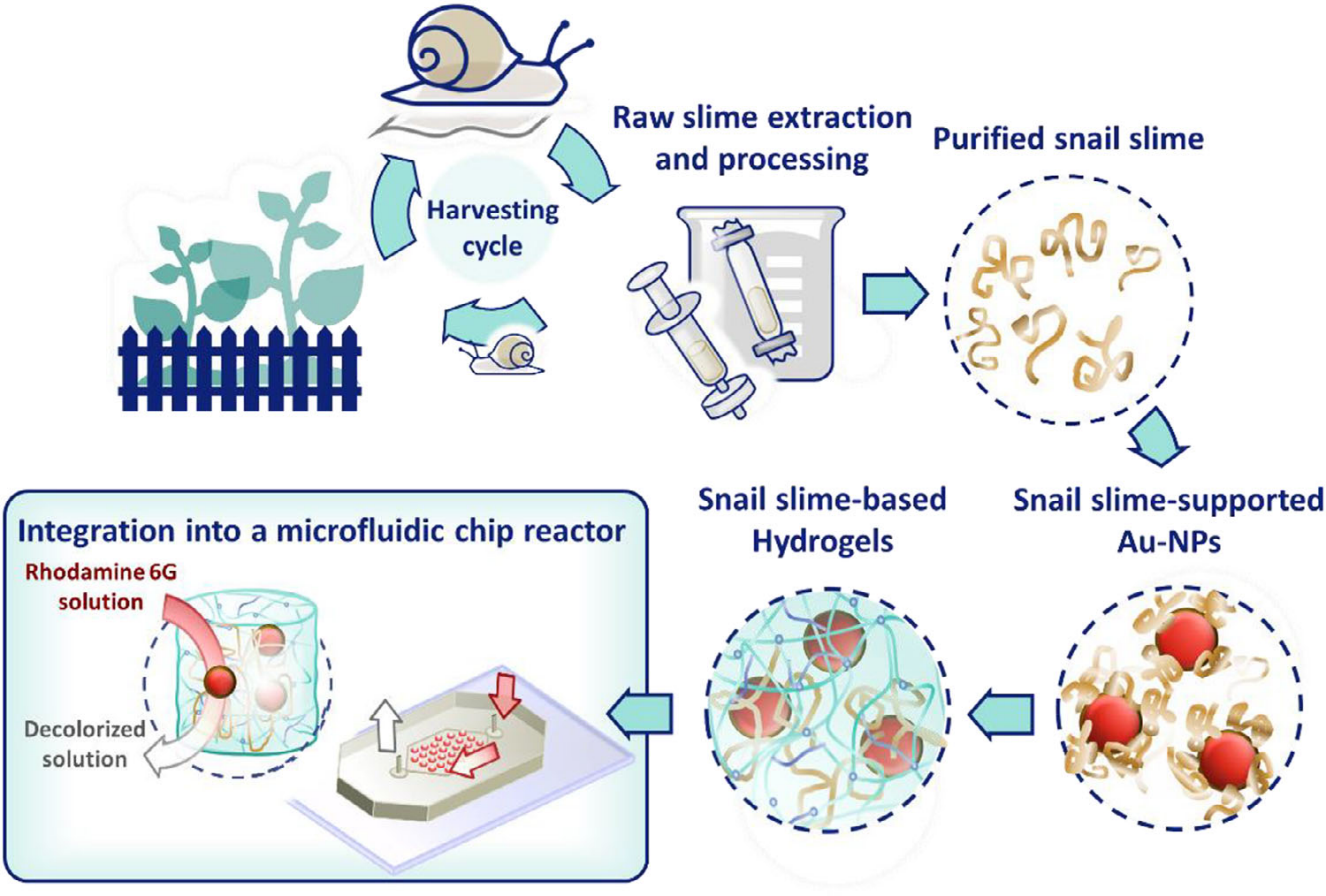
Project overview and optimized process as a step‐by‐step sketch: Raw slime extraction in a harvesting cycle, processing to purified snail slime (top) to the production of slime‐supported Au‐NPs and slime‐based nanoparticle‐comprising hydrogels for integration into a microfluidic chip reactor.

This work looks into the first application of snail‐slime as a material in microfluidics. Starting with the synthesis of snail slime‐supported Au‐NPs, these are then embedded into an environmentally friendly hydrogel matrix based on snail slime (Figure 1). Machine‐derived – i.e. industrial – snail slime from species *Helix aspersa* (*H. aspersa)* served as a base line and was compared to slime from a newly developed manual harvesting method to show the potential of a more animal‐friendly way to get the slime. These samples followed a newly established procedure of purification, nanoparticle synthesis, hydrogel dot preparation and integration into a microfluidic chip reactor for the oxidative degradation of Rhodamine 6G [46, 47, 48]. Snail slime would then act as chemical agent, stabilizing ingredient and chemical building block, i.e. be applied with three different functions. We thus developed a microfluidic flow reactor, utilizing the catalytical and antibiotic activity of snail slime‐based hydrogels with Au‐NPs.

## 2 Results and Discussion

**FIGURE 2:**
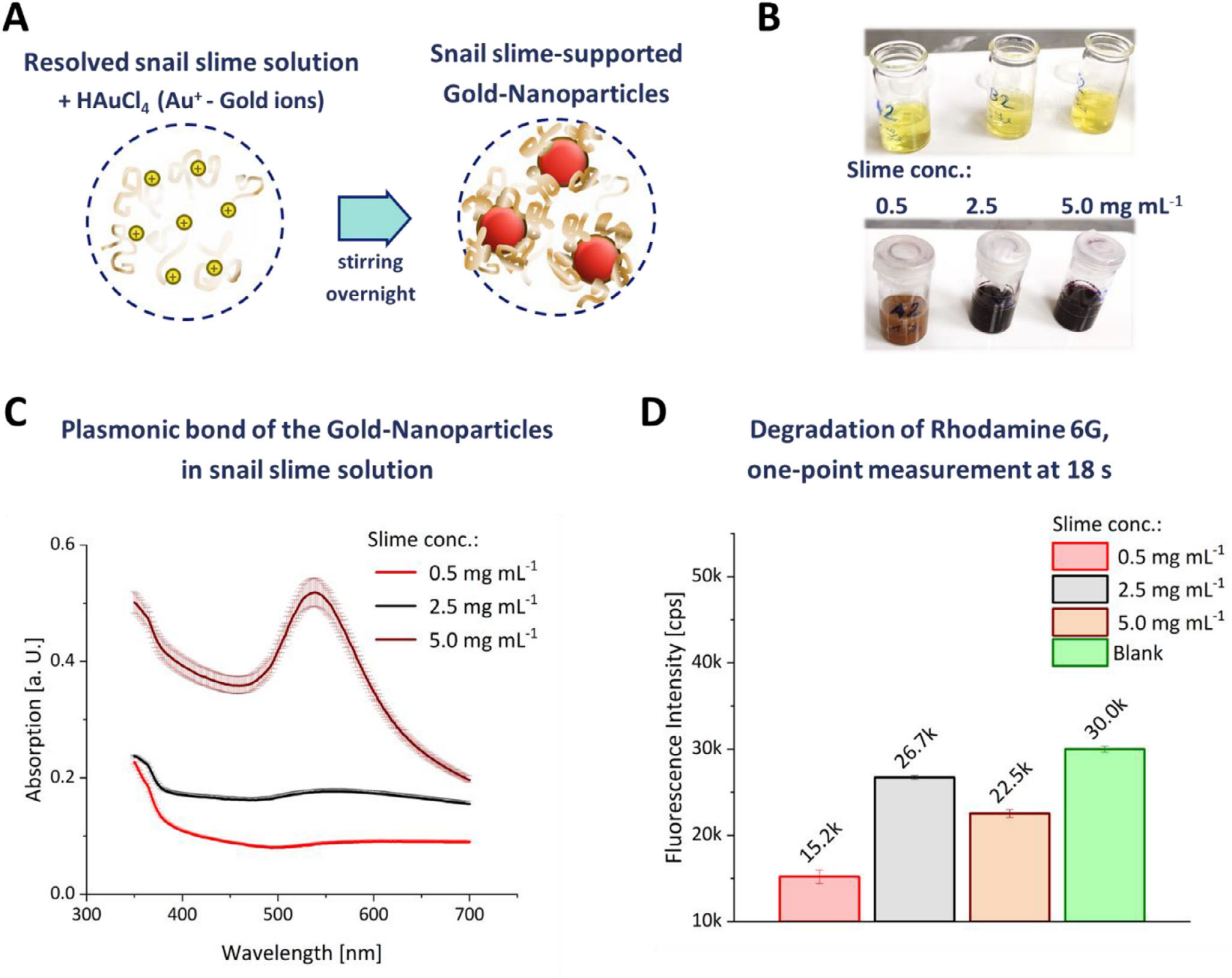
Nanoparticle synthesis and initial characterization by UV–vis spectroscopy, activity experiments, originating from various concentrations of snail slime: (A) Schematic illustration of the preparation of Snail slime‐supported gold NPs, by typically mixing a solution of purified, resolved snail slime 1: 1 with 10 mM precursor solution HAuCl_4_ for final snail slime concentrations of 0.5/2.5/5.0 mg mL^−1^; (B) Color change due to nanoparticle formation from yellow to red (0.5 mg mL^−1^), black (2.5 mg mL^−1^) and brown (5.0 mg mL^−1^); (C) UV/VIS spectra of Au‐NPs, obtained from different snail slime concentrations, including the characteristic plasmon bond in a range of 530 to 550 nm; (D) Relative decline in fluorescence intensity of Rhodamine 6G in the presence of the Au‐NP/slime solutions after 18 s (Figure S1B for entire period of 120 s), (excitation wavelength of 478 nm and emission wavelength of 556 nm).

The nanoparticle synthesis was derived from Di Filippo et al. [17]. Therefore, 4 mg purified and freeze‐dried industrial snail slime was resolved in 2 mL MilliQ water, and mixed 1:1 with a freshly prepared 10 mM precursor solution of HAuCl_4_. Afterwards, the solution was left stirring overnight at room temperature under the exclusion of light. The following day, a significant change in color of the snail slime solution from yellow to brick‐red, violet or black for snail slime concentrations of 0.5, 2.5, and 5.0 mg mL^−1^, respectively, occurred as a clear indication of the presence of Au‐NPs or nanoclusters (Figure 2B).

**FIGURE 3:**
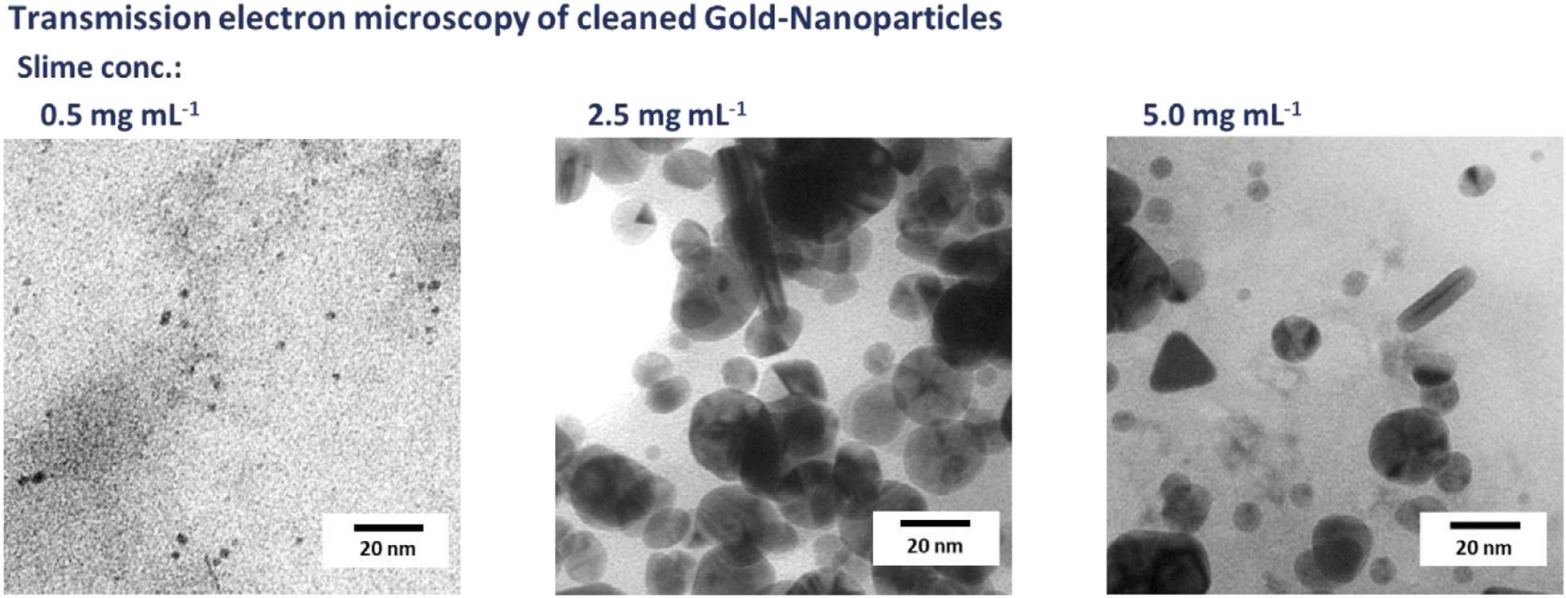
Transmission electron microscopy (TEM) images presenting smaller scaled, spheric NPs (0.5 mg mL^−1^/left) as well as large clusters and multiple geometric shapes (2.5 mg mL^−1^/center) and both manifestations in combination (5.0 mg mL^−1^/right).

NPs are generally characterized by striking catalytic activity, due to the size quantization effect, i.e. the high ratio of active surface to passive volume [22, 23, 24]. Resulting from the increased surface‐to‐charge ratio of the surface plasmon, a higher photon energy for excitation is required, resulting in absorption of light in the visible range (= surface plasmon resonance, SPR). Since higher intensity of the SPR means higher yield of NPs, UV–vis spectroscopy represents a suitable technique for monitoring and evaluating the synthesis of the Au‐NPs. Analyses of the snail slime‐supported Au‐NPs exhibited the expected intense plasmon bonds around 530 to 550 nm, especially at a high concentration of 5.0 mg mL^−1^ snail slime (Figure 2C, brown line). Even though snail slime concentrations of 0.5 and 2.5 mg mL^−1^ resulted in the mentioned coloring, their absorption spectra did not show the characteristic SPR band, likely due to insufficient concentration of non‐agglomerated NPs. The presence of gold NPs was still clear from the colored solution and confirmed later by electron microscopy (Figure 3; Figure S2). A higher yield of NPs was hypothesized to lead to an overall higher catalytic activity, which was checked by the catalytically accelerated degradation of Rhodamine 6G.

Using pre‐optimized activity measurements inspired by Ramakrishna et al., [48] the measurements were performed by adding 20 µL 5.0 mM NaBH_4_ solution as a reduction agent and 10 µL nanoparticle solution to 20 µL 0.1 mM Rhodamine 6G within a 96‐well microplate. Since the reaction proceeded rapidly, the degradation of the dye and thus the decrease in fluorescence intensity was determined by comparison against a reference solution without the presence of catalytically active NPs (Figure 2D). All measurements were conducted using an excitation wavelength of 478 nm, an emission wavelength of 556 nm and all results were compared at t = 18 s.

Much to our surprise, the sample originating from the lowest amount of slime, i.e. the least SPR absorption intensity, presented the strongest drop in fluorescence intensity by nearly 50% compared to the blank value without nanoparticle solution. In contrast, the highest concentration of snail lime, which led to the highest yield of NPs achieved a decrease of 25%, while the medium concentration realized a decrease of only 11% (Figure 2D). This observation at 18 s is representative for the entire time period of 120 s (Figure S1B).

A possible explanation of this behavior is that the deciding factor for catalytic activity is not the amount of NPs, but the amount of accessible surface area. In other words, both, the nanoscale size and the homogeneous distribution of the particles in solution play an important role. By comparing the TEM images of the different Au‐NPs, it became clear that high concentrations of the stabilizing agent, i.e. 2.5 instead of 0.5 mg mL^−1^ snail slime, promote the formation of larger, multi‐shaped NPs and nanocrystals as well as agglomerates, i.e. reducing the surface area that is available for catalytic activity (Figure 3; Figure S2). These images provided clear evidence for the formation of NPs, but agglomerates of the NPs in the images prohibited a more refined size analysis to provide an overview of the formed sizes. Further increasing the concentration to 5.0 mg mL^−1^ snail slime then increases the number of such clusters and hence increased the catalytic activity again. Since the catalytic activity of 0.5 mg mL^−1^ was highest in this series, but not necessarily the maximum, the most effective snail slime concentration was sought for as the next step.

The overall details of the optimization process of the reaction conditions as well as snail slime concentration are comprised in the Supporting information (Table S1, Figures S3 and S4). By applying various amounts of industrial snail slime in the range from 0.2 to 4.0 mg mL^−1^ several batches of Au‐NPs were prepared as mentioned above. The following fluorescence evolution over 5, 10, and 20 min showed that the most active NPs (= strongest decline in fluorescence) was noted at 1.0 mg mL^−1^ snail slime with a degradation of 93.9% over a measurement period of 20 min. Since the values for other low concentrations like 0.8 (93.8% decline) and 0.6 mg mL^−1^ (93.5% decline) were close, we opted to remain at 0.5 mg mL^−1^ snail slime concentration for the following proof‐of‐concept experiments.

**FIGURE 4:**
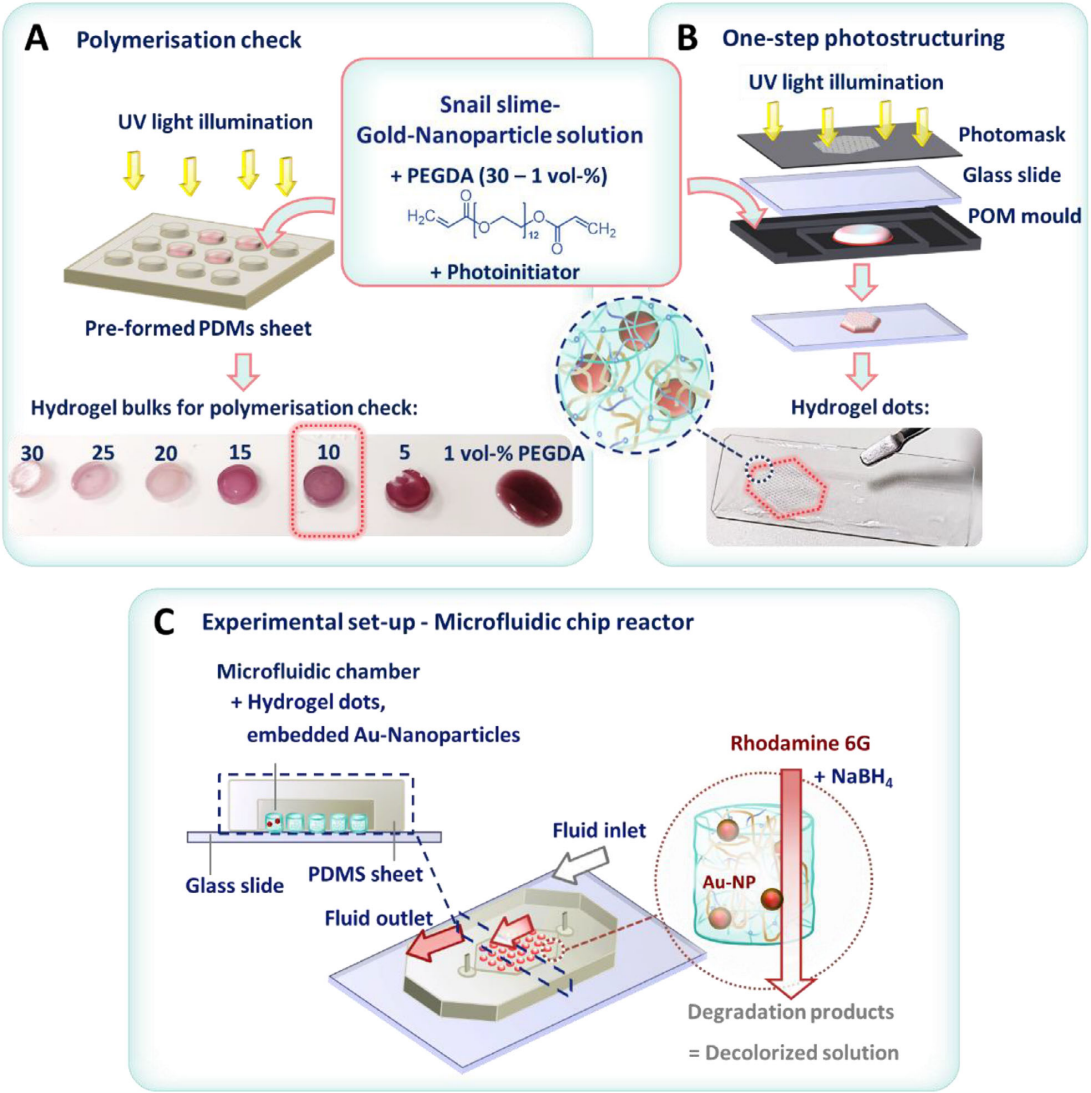
Photopolymerization strategies for manufacturing of snail slime‐based gold nanoparticle hydrogel structures for integration into a microfluidic chip reactor: (A) Polymerization check for evaluating the ability to form stable bulk hydrogels in the range from 30 to 1 vol.% cross‐linker PEGDA; (B) One‐step photostructuring of microscale hydrogel dots arrays for implementation into a microfluidic single‐chamber reactor (based on previously published results) [45, 49]. (C) Structure of the microfluidic chip reactor for performing degradation of the Rhodamine 6G by snail slime‐based Au‐NPs, encapsulated in hydrogel dots under fluid‐flow conditions.

The utilization of snail slime‐supported Au‐NPs in a microfluidic environment requires their immobilization on or in suitable carrier materials, for instance highly porous hydrogel networks. Based on slime proteins and the artificial cross‐linker Poly(ethylene glycol) diacrylate (PEGDA) we enabled the formation of 3D hydrogel networks for in situ incorporation of previously synthesized Au‐NPs (Figure 4). Derived from a precursor composition for the physically entrapment of enzyme molecules [45, 49, 50], multiple approaches containing different ratios of snail slime‐comprising Au‐NP solution, cross‐linker and photoinitiator were assessed. (Table S2) Evaluating them for their ability to form stable macroscale bulk hydrogels showed that 10 vol‐% PEGDA was the most suitable concentration for the synthesis of stable, dark red bulk hydrogel (Figure 4A). Less PEGDA resulted in instable gels and more densely cross‐linked gels showed a decoloration and were hence not considered further for this reason. It should be noted that the snail slime worked as a reducing agent on the Au^3+^ solution, stabilizing agent on the Au‐NPs and chemical building block for the hydrogel dots, showcasing its’ remarkable chemical versatility.

The optimized precursor composition was subsequently applied for the synthesis of micro‐scale hydrogel dots arrays for microfluidic reaction chambers (Figure 4B; Figure S5). An established procedure of one‐step photo‐structuring and implementation into a microfluidic chip reactor was carried out using an established protocol (Figure 4C) [49].

**FIGURE 5:**
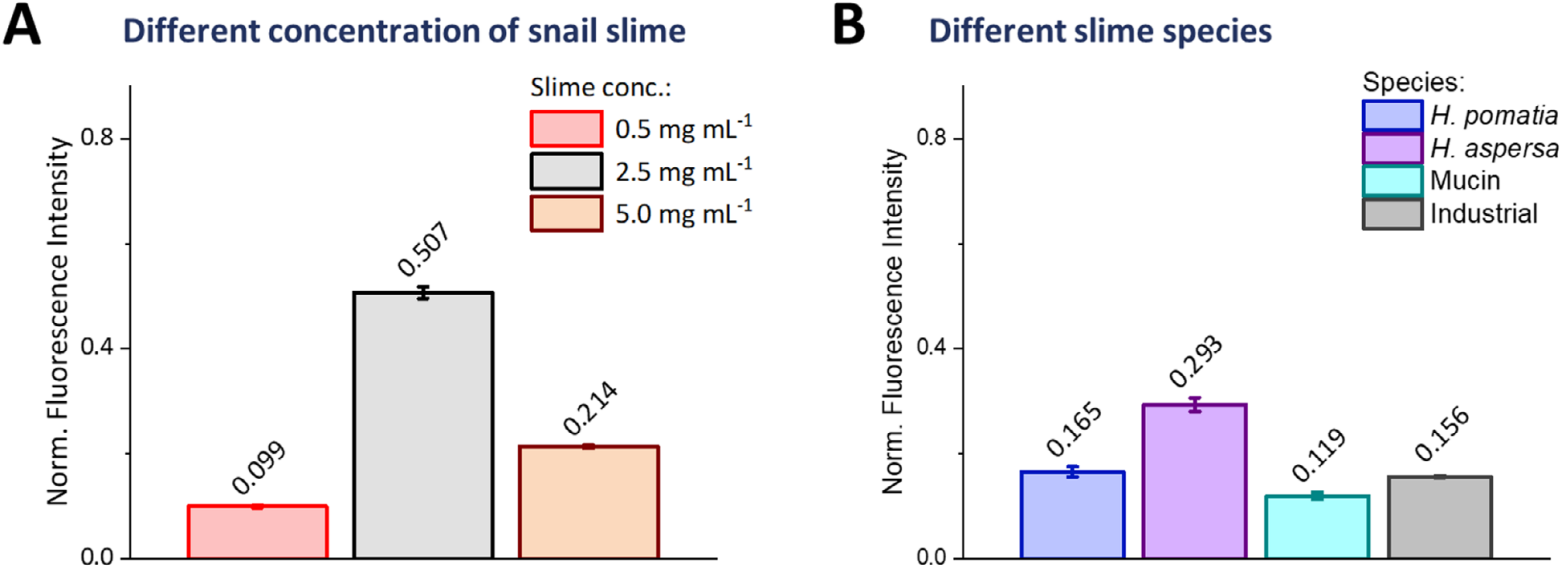
Fluorescence intensity examinations for comparison the ability of different slime concentrations (industrial extracted slime from *H. aspersa*) and various slime species for the fabrication and physically entrapment of Au‐NPs into hydrogel dots arrays and implementation into a microfluidic chip reactor: (A) Fluorescence intensity at 120 min for the adapted snail slime concentrations of 0.5, 2.5, and 5.0 mg mL^−1^, introduced in Figure 2; (B) Fluorescence intensity at 120 min for manually harvested slime from *H. aspersa* and *H. pomatia*, Mucin and industrially extracted snail slime; samples were collected at the outlet of the microfluidic chip as a triplet at an excitation wavelength of 478 nm and an emission wavelength of 556 nm and finally normalized to the respective start values (0 min).

Pure snail slime‐based bulk hydrogels and hydrogel dots without Au‐NPs have been manufactured as well (Table S3). These networks demanded lower quantities of photoinitiator and cross‐linker since no proteins were required to stabilize the Au‐NPs. Other than that, no notable differences were observed and this important control gel was now also available. In order to construct the final chip, the glass substrate bearing the snail slime‐based, Au‐NP‐comprising hydrogel dots was cleaned alongside the performed single‐chamber‐PDMS sheet. The dried PDMS chamber was gently placed on the glass slides for incorporating the hydrogel dots and the overall system was enclosed with an aluminum holder for stabilization, avoiding leakages and detachment of PDMS and glass surface (Figure 4C) [45]. The reaction solution containing Rhodamine 6G was supplied and removed at a consistent flow of 10 µL min^−1^ through polytetrafluoroethylene (PTFE) tubes. The microfluidic chip reactor is visualized in Figure 4C, the complete setup is included in the supporting information (Figures S6 and S7). With the system in‐hand, the microfluidic chip reactor was built (Figure 5A) and flushed for 2 h with the intensely colored reaction solution of 0.01 mM Rhodamine 6G and 0.5 mM NaBH_4_. Please note that the concentration of Rhodamine 6G had been reduced in comparison to the experiments in solution to ensure reduction of the dye in the flow as well. The complete emission spectra were recorded in 10 min intervals from the start until 120 min. For the concentration of 0.5 mg mL^−1^ snail slime during the nanoparticle synthesis (Figure S8A), a significant discoloration of the solution could be already recognized within the first 10 min, while the Rhodamine 6G associated emission maximum around 555 nm nearly vanished over the entire measuring period. In accordance with the data on the stabilized free Au‐NPs, the specimen of 0.5 mg mL^−1^ was able to diminish the Rhodamine 6G level more effectively and over a longer period of time in comparison to samples from 2.5 and 5.0 mg mL^−1^ snail slime (Figure S8B). More specifically, after 120 min a drop by 90% in relation to the initial fluorescence intensity was realized for 0.5 mg mL^−1^ snail slime, while 2.5 mg mL^−1^ enabled a decrease by merely 49% and 5.0 mg mL^−1^ by 79%.

The Au‐NPs hence retained their catalytic activity at low concentrations of snail slime in the microfluidic system and were even stabilized and usable for significantly longer than unbound, i. e. under stationary conditions (Figure 2D). Immobilizing active NPs in the microfluidic system laid the basis for further chemical reactions under mild conditions. These include, for example the oxidation of alcohols and alkanes [51], selected C‐C coupling [52], reduction reactions [53] and multiple degradation reactions [48]. Additionally, biosensing applications may be realized [34, 54, 55].

Finally, the results of our proof‐of‐concept experiments were transferred to snail slime from different harvesting methods, different snail species and the Mucin structural protein as reference. Within this series, the following samples were selected: manually collected slime from *H. aspersa* to check for a possible influence of the harvesting method, similarly collected slime from *H. pomatia* to check for the snail species and controls of artificial synthesized structural protein Mucin and the industrially extracted snail slime from *H. aspersa*, which was utilized for all previous experiments.

Au‐NPs and nanoparticle‐comprising microscale hydrogel dots of each sample were synthesized and characterized using the procedures described above. In addition, the activity measurements using the degradation of Rhodamine 6G of the individual samples were performed according to the methodology just discussed, always using 0.5 mg mL^−1^ snail slime to produce and hold the Au‐NPs in the hydrogel dots.

At an initial glance, all four slime variants presented similar reaction processing, with a rapid decrease in fluorescence intensity over time, followed by negligible low regeneration and equilibrium adjustment (Figure S9C). Being reproducible at this variety is an unexpected strong point of this natural product. In comparing the fluorescence intensities normalized to each initial value, it became notable that the manually harvested slime from of *H. aspersa* showed a slightly lower catalytic activity (29.3% remaining fluorescence), than the industrially collected slime (15.6% remaining fluorescence). However, since the manual method was not optimized, this could be expected. It should be noted that the difference was small enough, that minor adjustments in the reaction in the harvesting conditions could led to a clear shift in favor of the manually – animal‐friendly – harvested slime. It was in this regard a positive surprise that the manually collected slime from *H. pomatia* was more active (16.5% remaining fluorescence). Since this snail species is the one native to the point of collection (Sachsen‐Anhalt, Germany), these ones are adapted best and for that reason may produce the catalytically most active slime. This now serves as an important first lead and the exact components that promote this observation will be at the core of future studies. As a positive control the pure Mucin was tested as well and produced the most active Au‐NP‐hydrogels for the microfluidic device (11.9% remaining fluorescence, Figure 5B).

Following the successful implementation of the snail slime in this context, the chemical background needs to be looked into. Following the extraction methods and slime species, it can be assumed that the structural protein group of Mucins, as described by Cerullo et al., [56] are the key component responsible for NP formation and hydrogel synthesis. These long‐chain glycoproteins are composed of a central protein chain, which alternately exhibits domains of serine/threonine/proline and cysteine, as well as branched glycan side chains [57, 58], comprising multiple sugar units. These include galactose, *N*‐acetylgalactosamine, mannose, *N*‐acetylglucosamine, fucose, xylose, *N*‐acetylneuraminic acid and *N*‐glycolylneuraminic acid [56, 59]. Among these, cysteine is a likely reduction agent in the context of protein‐assisted NP synthesis [60, 61]. The reduction of the introduced gold ions (Au^3+^) to metallic gold atoms (Au^0^) was established due to electron transfer originating from the thiol group.

Following covalent and electrostatic interactions between the oligosaccharide‐side chains or cysteine residues and the gold NPs, Mucin chains were subsequently likely adsorbed onto the NP surface. Following this hypothesis, Mucin proteins functioned as a reduction agent as well as stabilizing and capping agent [61, 62, 63]. This would lead to the conclusion that the slime from *H. aspersa* had a higher cysteine content, leading to a higher reactivity in this project. While consistent with literature, these hypotheses need to be tested further in the future.

Although the reference material applied in this publication was obtained from vertebrates, i.e. porcine stomach lining, Mucins in and on the surface of snails and higher organisms fulfil the same functions of retaining moisture, providing protection and mediating adhesion. Despite the variations in the structural complexity of the protein backbone and the glycogen‐side chains, the basic structure allowed for a comparison of these materials and they did present a similar behavior during the Au‐NP formation and hydrogel synthesis [64, 65, 66].

Hydrogel formation was performed using a well‐established procedure for one‐step photo‐structuring. The common photoinitiator LAP was activated by UV irradiation, initiating a free radical polymerization involving Mucin oligomers and the cross‐linker PEGDA. It means that covalent bonds were formed between the glycogen‐side chains of the Mucin and the acrylate group of PEGDA [67, 68, 69], although the exact reaction mechanism and hence the exact network structure is not fully understood. UV‐light irradiation causes the photoinitiator to split and form radicals, but it can also cause changes in the Mucin structure, leading to radical formation and cross‐linking [70, 71].

As we utilized snail slime as a natural, inhomogeneous product in this work, the degree of purity affects the reproducibility for future application‐oriented research. For industrial snail slime, the results of quality control are documented in a technical data sheet. The recorded protein content amounts to 0.05–0.2 g per 100 g slime (0.05–0.2 wt.%) and the mucopolysaccharide content, which serves as an indicator of Mucin concentration, amounts to 1.0–2.0 wt.% [72]. This industrial sample is hence viewed as reproducible. Regarding the manually harvested slime from *H. aspersa* and *H. pomatia*, the degree of purity and the amount of Mucin are crucial, but complex parameters to handle. Following the proof‐of‐concept in this work, defined analytical procedures are required in the next stages to clearly quantify the composition. To ensure the reproducibility of our results and keeping the mucus composition as homogeneous as possible, we established consistent husbandry and feeding conditions and developed an animal‐friendly methodology for slime harvesting.

## 3 Conclusions

Within the scope of our research, we have tapped into chemical potential of snail slime. In a process based entirely on the interaction of slime‐specific proteins, it acted as reducing and stabilizing agent for Au‐NPs and chemical builing block for hydrogels. Their application in hydrogel‐based microfluidics opened up a whole new range of applications for this kind of bioderived polymer.

These Au‐NP‐hydrogel composites were successfully adapted into microscale hydrogel dot arrays for integration into a microfluidic chip reactor and enabled chemical processes under fluid‐flow conditions with a currently unknown potential to be reused. Especially the catalytic activity of the snail slime‐based Au‐NP‐hydrogels was verified and quantified by monitoring the degradation of the Rhodamine 6G dye within the microfluidic setup. While industrially extracted snail slime from *H. aspersa* served as a reference material, manually harvested slime from H. aspersa, H. pomatia and Mucin showed that our acid‐free (i.e. animal‐friendly) way to harvest slime actually leads to a chemically (higher reactivity) more valuable material. In addition, the mild extraction method based on NaCl leads to less interference with analytical methods and can enable more advanced applications in the long term. The combination of directly quantifiable dye degradation and the renerative nature of the starting material, the fabricated functional hydrogel‐on‐chip microfluidic devices ready the way for the development of future lab‐on‐chip devices based on snail slime from *H. aspersa* or *H. pomatia*. The protein‐hybrid hydrogels provide the structural flexibility and chemical properties for future diagnostic or medical applications. In this way, our studies created a broad range of new options for the efficient and creative utilization of natural regenerative resources by fabricating naturally modified hydrogels and the development of sustainable lab‐on‐chip devices for rapid tests or continous monitoring. The Au‐NPs embedded in protein‐based hydrogel structures within a microfluidic chip reactor are not restricted to the decolorization of Rhodamine 6G, but may be adapted for further oxidation reactions and multiple degradation reactions. Given its micro‐structured design, the microfluidic chip reactor demonstrates a high degree of flexibility with regard to reaction conditions, which can now be explored in future studies.

## 4 Experimental Section

### 4.1 Materials

Snail slime from *H. aspersa* and *H. pomatia* was collected manually from individuals, provided by Schneckenzucht Altmark GbR. Machine‐extracted “industrial” snail slime was ordered from Lumacheria Italiana srl. Sodium Chloride (NaCl, Ph. Eur.) was obtained from KMF Laborchemie Handels GmbH. Rhodamine 6G (for microscopy) and Lithium phenyl‐2,4,6‐trimethylbenzoyl) phosphinate (LAP, ≥95%), was purchased from Merck KGaA. Chloroauric acid (HAuCl_4_ * 3 H_2_O, ≥99.9% trace metals basis), Sodium borohydride (NaBH_4_, ReagentPlus, 99%) and Poly(ethylene glycol) diacrylate (PEGDA, M_w_ 525 g mol^−1^) were purchased from Sigma‐Aldrich. Mucin (75%–95%, for biochemistry) was received from Carl Roth. Isopropyl alcohol (IPA, extra dry, 99.5%) was ordered from Acros Organics. Microplates (Greiner 96 well plates, polystyrene) and microplate lids (Greiner multiwell plate lids) were provided from Sigma‐Aldrich. Glass slides (Menzelglas, Thermo Scientific, 26 × 76 × 1 mm^3^) were purchased from Merck KGaA. Pasteur pipettes (graded, max. 3,4 mL, LDPE), centrifugal tubes CELLSTAR (transparent, 50 mL), CHROMAFIL ‐Syringe filters (PES, pore size 5.0 µm) as well as Dialysis membrane Spectra/Por 7 (MWCO, 1,000 Da) were ordered from Carl Roth. Purified water (MilliQ) was reached by using a Milli‐Q Integral 5 system from Merck Millipore.

### 4.2 Snail Slime Processing

For manually harvesting of snail slime 20 individuals were collected from the parcels, cleaned with water and placed in a stainless steel laboratory dish. Each snail was three times sprayed with 3%‐NaCl solution and in between left to move on the plate for 5 min. Afterwards the animals were washed with water and returned to the fields. The snail trails on the plate were collected using pasteur pipettes and wipers and were stored for transportation in centrifuge tubes.

The pH value was determined for machine‐extracted as well as manually harvested snail slime and adjusted in a range from 7.0 to 8.0 by adding 1 M NaOH. For pH monitoring HI 83141 pH meter with pH electrode (HANNA instruments, pH 0…14 ± 0.01 pH, mV±1999 ±0.2 mV (ISE) and ±1 mV (ORP), °C 0.0 to 1000.0 ±0.5°C, Ag/AgCl, glass‐combination) were used. Afterwards the samples were filtrated using PES‐Syringe filters (pore size 5.0 µm) and separated in 40 mL portions. Each approach was filled into 20 cm MWCO dialyses membrane (1000 Da) and placed in 2L‐beakers with deionized overnight. The next day that water was replaced two times with MilliQ water for 2 h each. Finally, the purified samples were transferred into 250 mL round‐bottom flasks and pre‐frozen in the freezer before attached to an Alpha 1–4 LDplus freeze‐dryer from CHRIST.

### 4.3 Nanoparticle Preparation

4 mg purified and freeze‐dried snail slime were resolved under stirring in 2 mL MilliQ water for 15 min. 2 mL freshly prepared 10 mM HAuCl_4_ in MilliQ water was added and the mixture stirred over night at room temperature under exclusion of light. The finished samples were checked for nanoparticle presence applying UV–vis spectroscopy to a 1:10 diluted sample

For perfoming activity measurments 1 mL nanoparticle solution was centrifugated for 10 min at 15 000 rpm and two times washed with MilliQ water, using the MiniSpin Plus centrifuge from Eppendorf (14 100 × g, 800–14 500 rpm).

Afterwards 20 µL cleaned nanoparticle solution was added to a mixture of 10 µL 10 mM NaBH_4_ and 20 µL 0.1 mM Rhodamine 6G in 150 µL MilliQ directly in a well of a Greiner Microplate (96 well, flat bottom transparent, PS) and monitored at an excitation wavelength of 478 nm and an emission wavelength of 556 nm using microplate reader (Infinite 200 Pro – Series Infinite M Nano+, Tecan Group Ltd. Männerdorf, Switzerland) and Tecan i‐control software.

### 4.4 Hydrogel Precursor Preparation

For preparation of the hydrogel precursor solution, 0.5 mL snail slime‐nanoparticle solution were mixed with 55.6 µL cross‐linker Poly(ethylene glycol) diacrylate (PEGDA) and 11.6 mg Lithium Phenyl‐2,4,6‐trimethylbenzoylphosphinate (LAP) and stirred for 1 h under exclusion of light at room temperature. The hydrogel bulks were fabricated by transferring 200 µL precursor solution to the preformed PDMS sheet (diameter cylindric bulg: 10 mm) and placed with a distance of 8 cm to the UV lamp and illuminated for 7.5 s using a Omnicure S2000 system (Lumen Dynamics Group Inc., Canada) equipped with a high‐pressure mercury lamp (0.35 W cm^−2^, emission spectrum: 320 – 500 nm). The hydrogel dots were manufactured by dropping 100 µL precursor solution into the single chamber POM mould, covering with a glass substrate and pre‐structured photo mask. The mould was placed with a distance of 8 cm to the UV lamp and illuminated for 7.5 s.

### 4.5 Microfluidic Experiments

The glass substrate bearing the hydrogel dots array, was rinsed with water and cleaned afterwards with isopropanol while avoiding contact with the hydrogel dots. The preformed single‐chamber‐PDMS sheet was first cleaned with a solution of non‐ionic surfactant and MilliQ water, second with ethanol. The dried PDMS chamber was gently placed on the glass slides for incorporation of the hydrogel dots, covered by an aluminum frame with an acrylic glass window on the top and enclosed into an aluminum holder.

Reaction solution of 500 µL 0.1 mM Rhodamine 6G and 250 µL 10 mM cooled NaBH_4_ solution in 4.25 mL MilliQ water was supplied and removed at consistent flow rate of 10 µL min^−1^ for 2 h, using an Asia syringe pump (Syrris Ltd, syringe volume: 50 and 100 µL, Royston, UK) through polytetrafluoroethylene (PTFE) tubes, attached to the inlet and outlet of the microfluidic system. The samples were collected at the outlet of the microlfuidic chip in 15 min steps and transferred to the microplate for fluorescence spectroscopy experiments using a microplate reader (Infinite 200 Pro – Series Infinite M Nano+, Tecan Group Ltd. Männerdorf, Switzerland) and Tecan i‐control software. For examination 30 µL sample were diluted with 90 µL MilliQ water and measured as triplet at an excitation wavelength of 478 nm and an emission wavelength of 556 nm in form of one‐point measurements and as required spectra in an emission range from 500 to 600 nm.

## Funding

This research was accomplished within the framework of the European Innovation Partnership “Agricultural productivity and sustainability”(EIP‐AGRI; 2022–24) of the European Agricultural Fund for Rural Development (ELER) in cooperation with Schneckenzucht Altmark GbR and the University of Applied Sciences Anhalt.

## Conflicts of Interest

The authors declare no conflicts of interest.

## Supporting information




**Supporting File**: marc70107‐sup‐0001‐SuppMat.pdf.

## Data Availability

The data that support the findings of this study are available from the corresponding author upon reasonable request.
