# Supplementary material for: Bio‐Based Microfluidics With Snail Slime: A By‐Product of Agriculture Plays an Exciting Role in the Chemistry of Microfluidic Reaction Chambers
Source: Macromol Rapid Commun. 2025 Oct 25;47(14):e00578. doi: 10.1002/marc.202500578 (PMC13384795; doi:10.1002/marc.202500578)
Supplement: Supplementary file 1 — Supporting File: marc70107‐sup‐0001‐SuppMat.pdf. [file MARC-47-e00578-s001.pdf]

Supporting Information

Bio-based microfluidics based on snail slime - a by-product of agriculture plays and active role in the chemistry of microfluidic reaction chambers.

Andrea Koball, Jens Gaitzsch**

1. Snail-slime supported nanoparticle synthesis

1.1. Influence of snail slime concentration on optical and activity properties

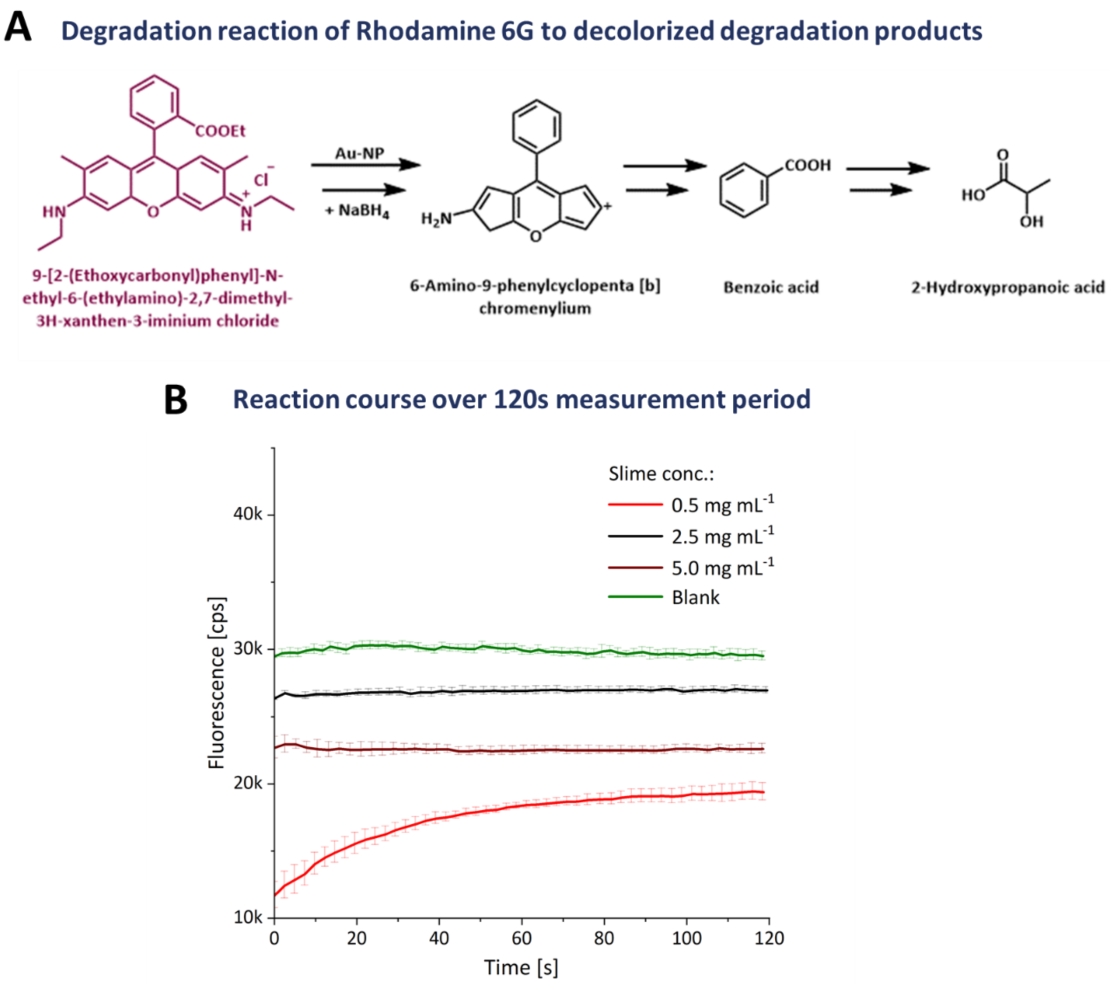

Figure S1. Time-resolved fluorescence monitoring of the catalytic activity by degradation of Rhodamine 6G: (A) Chemical transformation of Rhodamine 6G to colorless 6-Amino-9-phenylcyclopenta [b] chromenylium and the resulting decline in fluorescence intensity in comparison to reference solution without NPs (green): 10 µL nanoparticle solution was added

to a mixture of 20. µL 5.0 mM NaBH4, 20 µL 0.1 mM Rhodamine 6G and 150 µL in MilliQ water, applying an excitation wavelength of 478 nm and an emission wavelength of 556 nm.

The degradation of Rhodamine 6G was introduced in the main part for determining the catalytic activity of the manufactured snail slime-supported gold NPs.[1-3] Thereby Rhodamine 6G is decomposed to 6-Amino-9-phenylcyclopenta [b] chromenylium via a large number of intermediate steps, for which the mechanisms are not fully clarified (**Figure S1A**). An overview of the proposed mechanisms is presented by Rasheed et al.[2]
The procedure, which was orientated at Ramakrishna et al.,[3] was performed by mixing 0.5 mM $NaBH_4$ solution as a reduction agent, 0.01 mM Rhodamine 6G and nanoparticle solution within a 96 Well PES Microplate. The conversion and the color change took place immediately, thus the initial degradation process could not be fully observed. However, a clear degradation of the fluorescence intensity, using an excitation wavelength of 478 nm and an emission wavelength of 556 nm, can be seen in comparison to the nanoparticle-free approach. Subsequently, a chemical equilibrium was formed within the stationary system inside the microplate well, resulting in a gradual regeneration of the dye, increase in fluorescence intensity and formation of a plateau (**Figure S1B**).
As implied in the main section, the catalytic activity of snail slime supported gold NPs corrolated with their dimension and spatial strucutre. Additional to the presented TEM records at a magnification of 100,000 (**Figure 3)**, in **Figure S2A** images in a microscale range are shown, which underline the previous findings: lower concentrations of snail slime (0.5 mg mL^{-1}) resulted in small, homogeneous distributed NPs, while higher concentrations, increased the tendency to form various agglomerates, amorphous strucutures and nanocrystals. SEM-measurements, focusing on 0.5 mg mL^{-1} applied slime (**Figure S2B**), exhibit not only spheric and catalytic gold NPs, but a broad range of crystaline structures from rods and triangular, pentagonal plates up to polyhedrical modifications.

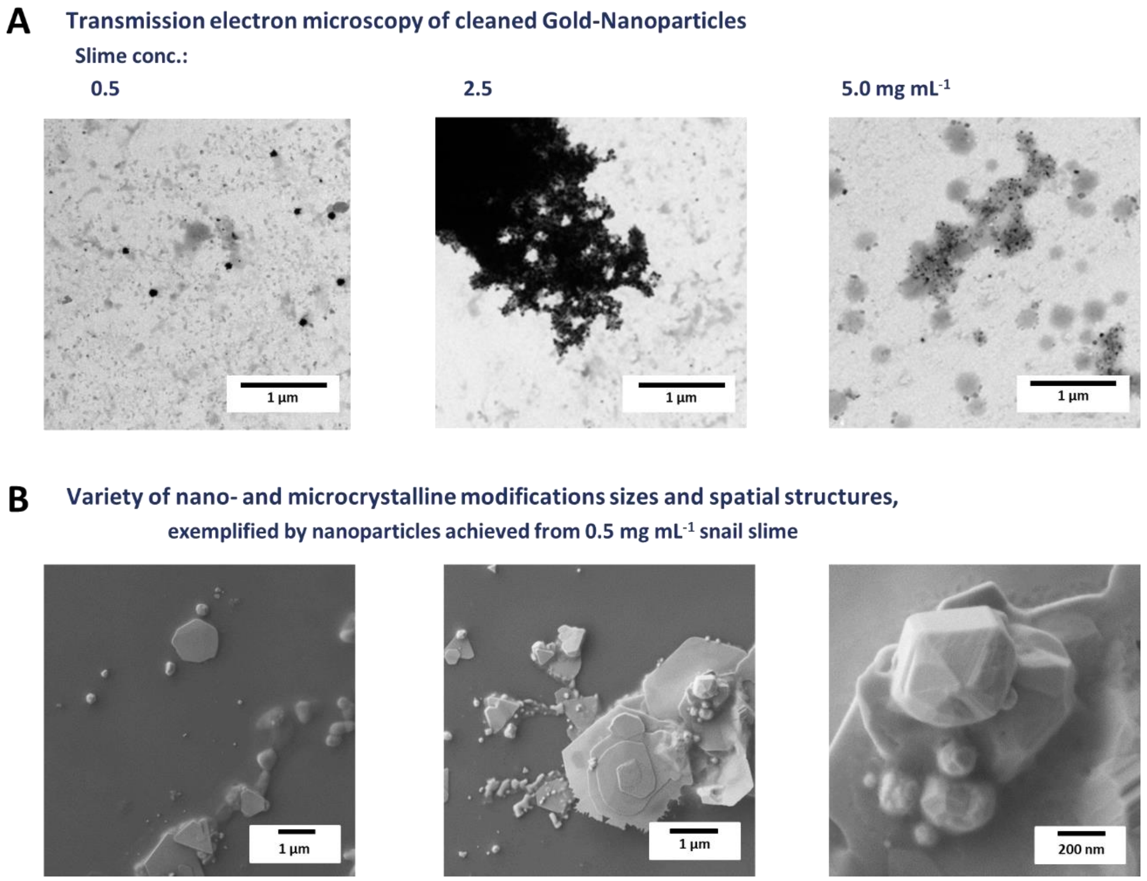

Figure S2. Visualisation of snail slime-supported gold NPs by TEM and SEM: (A) TEM images presenting smaller scaled, homogeneous distributed particles (0.5 mg mL^{-1}, left) as well as amorphic clusters (2.5 mg mL^{-1}, centre) and free, but slightly larger particles, connected by grey shaded slime residues (5.0 mg mL^{-1}right); measurements accomplished by Dr. Petr Formánek, using Libra 120 microscope (Carl Zeiss Microscopy GmbH, Oberkochen, Germany) at a magnitude 4,000-100,000x and an acceleration voltage of 120 kV; (B) SEM records of different locations within the sample achieved from 0.5 mg mL^{-1} snail slime, presenting versatile nano- and microcrystalline structures of rods, triangular and pentagonal plates and polyhedrical particles; measurements accomplished by Dr. Petr Formánek, using NEON 40 FIB-SEM microscope (Carl Zeiss Microscopy GmbH, Oberkochen, Germany) at a magnitude 24.0-100,000 x and an acceleration voltage of 1.0-10.0 kV.

1.2. Fine-Tuning of the reaction solution

Initial the reaction conditions have been optimized for enabling more accurate and long-term observation of the verification reaction of purified snail-slime supported gold NPs. Therefor the nanoparticle samples have been cleaned from remains of unconsumed snail slime and chloroauric acid by centrifugated as 1 mL batches for 10 min at 15,000 rpm and two times

washed with MilliQ water, using the MiniSpin® Plus centrifuge from Eppendorf® (14100 × g, 800-14500 rpm), before narrowed down to the original volume and concentration. Afterwards, Rhodamine 6G assays were performed by mixing various ratios of Rhodamine 6G, reduction agent $NaBH_4$, cleaned gold NPs and MilliQ water for a 200 µL solution directly in a well of a Greiner Microplate (96 well, flat bottom transparent, PS) as listed in in **Table S1**. The degradation reaction was monitored as a triplet for each approach at an excitation wavelength of 478 nm and an emission wavelength of 556 nm for 5/20 min, using microplate reader (Infinite® 200 Pro – Series Infinite M Nano+, Tecan Group Ltd. Männerdorf, Switzerland) and Tecan i-control software.

Table S1. Multiple approaches from starting conditions (‘Start’) to various optimizations (‘Opt-1/2/3/4/5), comprising different concentrations of Rhodamine 6G, $NaBH_4$ and nanoparticle solution in MilliQ water (vol-%), obtaining 200 µL batches each, for optimization of the reaction conditions for the degradation assay. The results are presented in **Figure S3**; in the beginning, i.e. during the ‘Start’ approach, NPs derived from three different concentrations of snail slime were applied: 0.1/0.5/1.0 mg mL^{-1}, for following approaches (Opt-1-5) only gold NPs from 1.0 mg mL^{-1} were applied.

	**Rhodamine 6G [mM]**	**$NaBH_4$ [mM]**	**Nanoparticle [vol-%]**
Start/**B**	0.1	0.25	5
Opt-1/**C**	0.1	0.50	5
Opt-2/**C**	0.1	1.0	5
Opt-3/**D**	0.01	1.0	5
Opt-4/**E**	0.01	0.50	5
Opt-5/**E**	0.01	0.50	10

In **Figure S3** the results of the overall optimisation procedure for achieving the most suitable reaction conditions by combining different ratios of Rhodamine 6 G, $NaBH_4$ and nanoparticle solution, are summarized. Thereby **Figure S3A** present the final fluorescence intensity values at 300 s (5 min) and 1200 s (20 min), normalized to the respective start value. Starting with a brief comparison of three operation concentrations of snail slime (0.1/0.5/1.0 mg mL^{-1}; **Figure S3B**), for the following steps gold NPs from 1.0 mg mL^{-1} slime were chosen. Successively, the concentration of $NaBH_4$ was adjusted (0.5 mM; **Figure S3C**) and the amount of Rhodamine

6G decreased (0.01 mM; **Figure S3D**). Not until the dye concentration was adjusted from Opt-2 to Opt-3, a distinct decline from 98.1 % to 50.6% was observed.

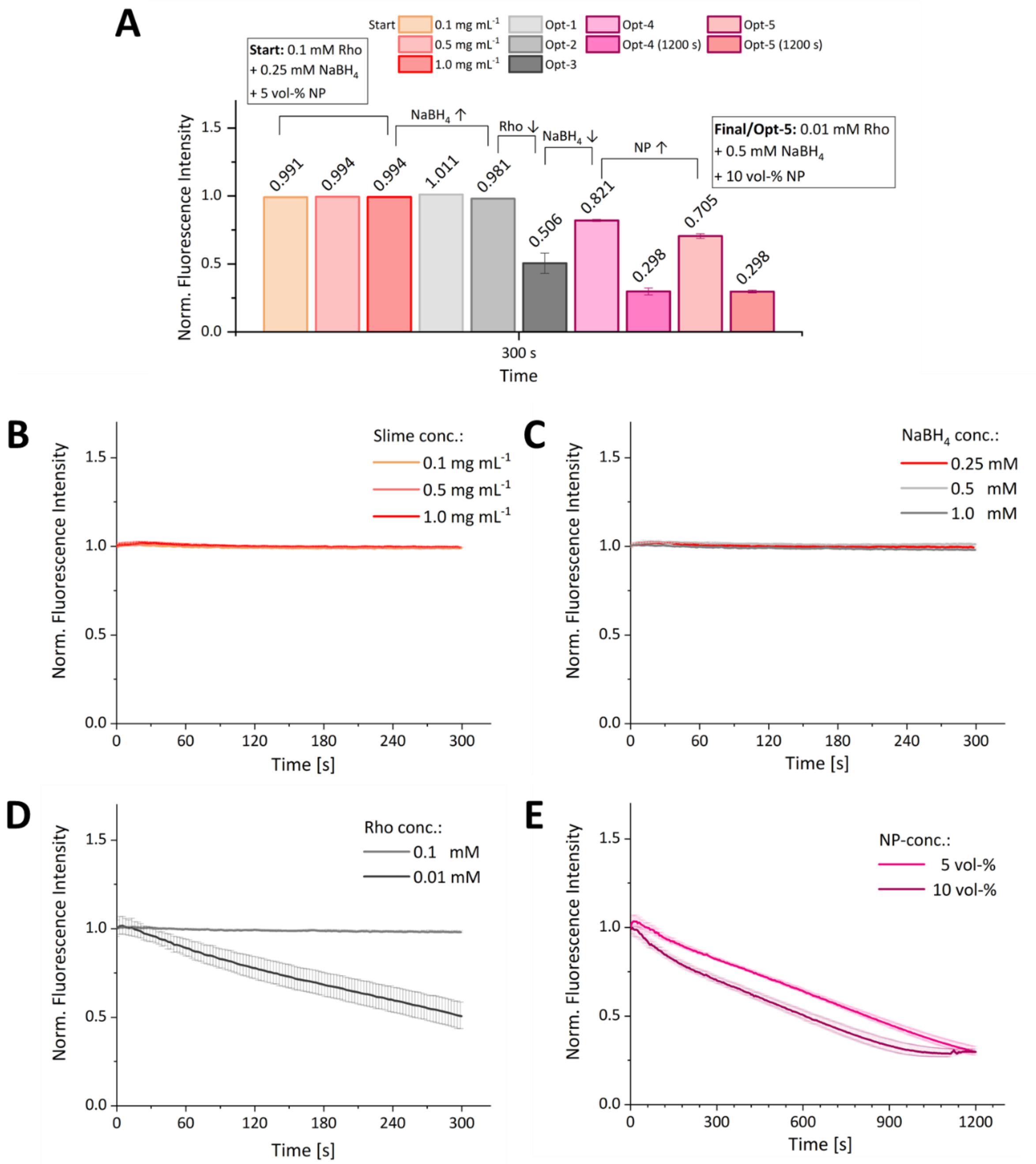

Figure S3. Results of the optimisation procedure, according to **Table S1**: (A) Normalized fluorescence intensity values at 300 s (5 min) and 1200 s (20 min), in relation to the respective start value, from 99% normalized fluorescence intensity at 0.1 mM Rhodamine 6G, 0.25 mM $NaBH_4$ and 5 vol-% nanoparticle solution at the beginning to finally 0.01 mM Rhodamine 6G, 0.5 mM $NaBH_4$ and 10 vol-% nanoparticle solution with 30% intensity after 20 min period; (B) Gold NPs, derived from multiple snail slime concentrations (0.1/0.5/1.0 mg mL^{-1}), applied for Rhodamine 6G degradation under start conditions; (C) Influence of the concentration (0.25/0.5/1.0 mM) of reduction agent $NaBH_4$ on dye degradation; (D) Impact of the

concentration (0.1/0.01 mM) of Rhodamine 6G on dye degradation; (E) Different amounts of nanoparticle solution (5/10 vol-%) with final concentrations of Rhodamine 6G and $NaBH_4$; monitored at an excitation wavelength of 478 nm and an emission wavelength of 556 nm.

At this point, the fit of $NaBH_4$ resulted in an increase of fluorescence intensity from Opt-3 to Opt-4 by 62%. While the amount of nanoparticle solution was set from 5 (Opt-4) to 10 vol-% (Opt-5), the resulting fluorescence intensity decreased from 82.1% to 70.5% at 300 s and both reached a plateau of 29.8% at 20 min (**Figure S3E**).

As a result, in the final approach over a period of 20 min, a comprehensible course of Rhodamine 6G degradation was received for comparing the influence of various snail slime concentrations on the catalytic activity of the prepared gold NPs (**Figure S4**).

1.2. Optimization of the snail slime concentration

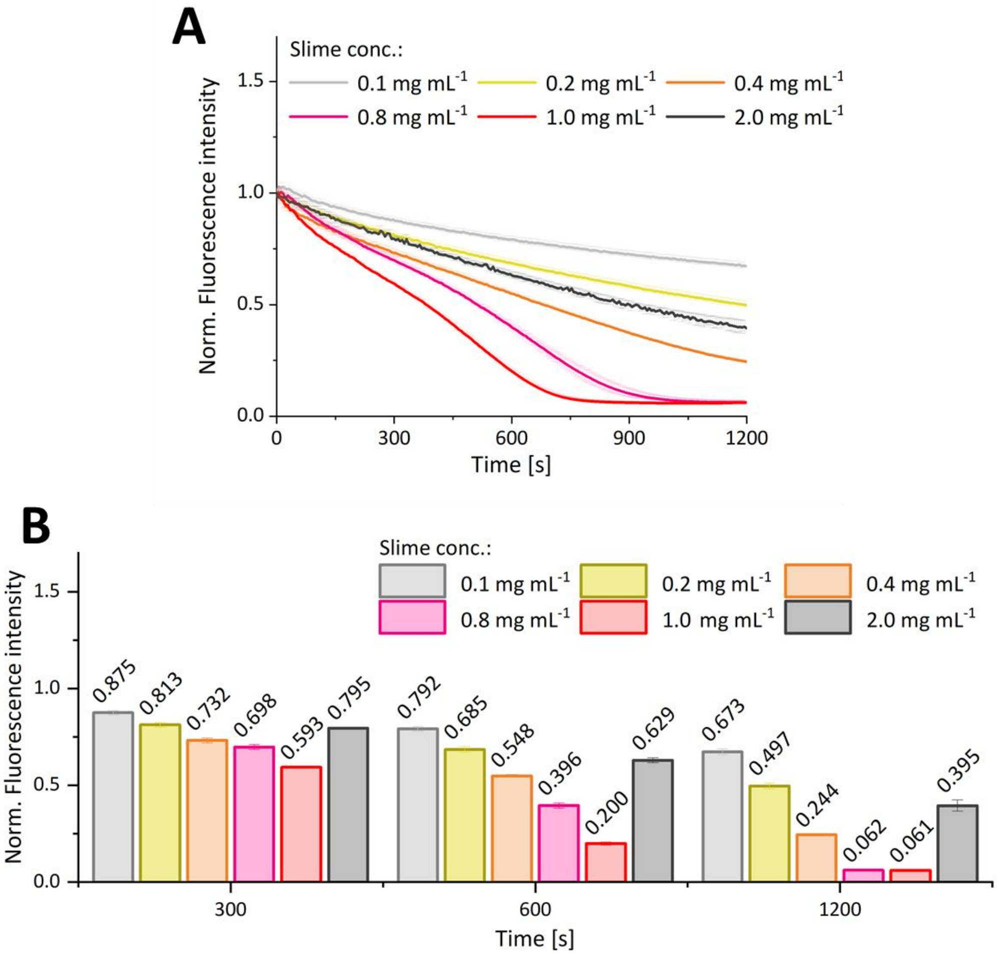

Figure S4. Fluorescence intensity measurements for determining the optimum snail slime concentration for manufacturing of the most catalytically favorable snail slime supported-gold NPs: (A) Fluorescence development over the total period of 20 min, normalized to the respective start value and (B) Normalized fluorescence intensity at specific times 5/10/20 min;

purified snail slime nanoparticle solution were mixed as 20 µL to a solution of 10 µL 10 mM $NaBH_4$ and 20 µL 0.1 mM Rhodamine 6G in 150 µL MilliQ water and monitored over 20 min under stationary conditions at an excitation wavelength of 478 nm and an emission wavelength of 556 nm.

By applying various amounts of industrial snail slime in the range from 0.2 mg mL^{-1} to 4.0 mg mL^{-1} several approaches of snail slime-supported NPs were prepared as mentioned above, by mixing 1 : 1 with 10 mM $HAuCl_4$ gold precursor solution and stirring overnight. Before carrying out the activity experiments, the centrifugation of the nanoparticle sample for 10 min at 15,000 rpm and two times washing with MilliQ water. According to the previous optimization results, 20 µL purified samples were added to a solution of 10 µL 10 mM $NaBH_4$ and 20 µL 0.1 mM Rhodamine 6G in 150 µL MilliQ water and monitored over 20 min at an excitation wavelength of 478 nm and an emission wavelength of 556 nm. The resulting fluorescence evolution over the entire period is shown in **Figure S4A** for a selection of nanoparticle samples, normalized to the respective initial value (0 min). The two lowest final concentrations of slime in the nanoparticle solution, 0.1 and 0.2 mg mL^{-1}, showed the least decrease in fluorescence intensity to a level of 67.3% and 49.7% at 20 min. As anticipated, the highest concentration of 2.0 mg mL^{-1} presented only slightly more degradation to an intensity of 39.5%. The strongest breakdown was realized by 1.0 mg mL^{-1} (6.1%), while the 0.8 mg mL^{-1} sample shows a slower decrease before closing up (6.2%). The influence of the different mucus concentrations on the activity of the prepared NPs stands out more clearly at a reaction time of 10 min. With exception of the maximum concenetration of 2.0 mg ml^{-1} the gradually decreasing fluorescence intensities behaved indirectly proportional to the amount of slime used: 79.2% for the lowest concentration of 0.1 mg mL^{-1}, followed by 68.5% (0.2 mg mL^{-1}), 54.8% (0.4 mg mL^{-1}) and 39.6% (0.8 mg mL^{-1}), down to 20.0% at 1.0 mg mL^{-1} (**Figure S4B**).

2. Snail slime-based, nanoparticle-comprising hydrogels

2.1. Optimization of the hydrogel precursor composition

As presented in **Figure 3A**, the capability of snail sime for manufacturing stable hydrogel networks was initial verified by simplification of an original hydrogel precursor composition for physically enzyme encapsulation[4] and stepwise decreasing of cross-linker content, as presented in **Table S2**. Thereby, the monomers 2-(Dimethylamino)ethyl methacrylate (DMAEMA) and 2-Hydroxyethyl methacrylate (HEMA) as well as MilliQ water were removed from the solution and replaced with slime-own proteins and nanoparticle-comprising solution.

In this context, three different snail slime concnetrations were applied, which however showed similar results regarding the abilit of hydrogel formation. As a result, a steady amount of photoinitiator Lithium Phenyl-2,4,6-trimethylbenzoylphosphinate (LAP) is required for hydrogel formation, while the proportion of cross-linker Poly(ethylene glycol) diacrylate (PEGDA) could be reduced to a third of the initial content.

Table S2. Several precursor compositions, derived from an original enzyme-comprising hydrogel, different ratios of cross-linker (PEGDA) to nanoparticle solution were applied for determining the lowest amount of cross-linker required, while manufacturing three-dimensional stable, nanoparticle-comprising hydrogel bulks and dots. PEGDA with a molar mass of 575 g mol^{-1} was applied for snail slime-based hydrogel structures, while the original precursor composition comprised PEGDA of 700 g mol^{-1}.

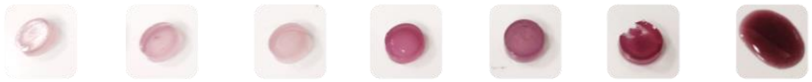

Original precursor composition[4]								
60.1 mol-% /32.3 vol-%	**Crosslinker** - PEGDA (700 g mol^{-1}/575 g mol^{-1})	**30 vol-%**	**25 vol-%**	**20 vol-%**	**15 vol-%**	**10 vol-%**	**5 vol-%**	**1 vol-%**
25.2 mol-%<br>14.7 mol-%	**Monomer** - DMAEMA, - HEMA							
39.9 mg	**Photoinitiator** - LAP	11.6 mg						
1.727 mL	**Solvent** - MilliQ water	0.5 mL nanoparticle solution (**0.4 mg mL^{-1}**)						
					0.5 mL nanoparticle solution (**8.0 mg mL^{-1}**)			
					0.5 mL nanoparticle solution (**5.0 mg mL^{-1}**)			
		Stable hydrogel bulks and dots					Unstable hydrogel bulks	No polymerization

2.2. Nanoparticle-free, snail slime-based hydrogel networks

In comparison, the utilization of pure snail slime solution without NPs allowed further reduction of applied cross-linker and photoinitiator, as presented in Table S3. In the process both components were examined separately. First, by using 11.6 mg photoinitiator, the smallest amount of PEGDA, which is required for the formation of stable hydrogel bulks and dots, was noted as 5 vol-%. Second, for a content of 10 vol-% cross-linker, nearly 2 mg LAP were identified as sufficient for hydrogel synthesis. This diminution of artificial ingredients compared to nanoparticle-containing slime solution is attributed to the fact that protein chains, which contributed to the reduction of the precursor and stabilization of the gold NPs, were modified in their chemical structure. In such a way deactivated, they were not able to participate in the hydrogel cross-linking process. In reverse, this also indicates that the hydrogel structures were clearly protein-based, i.e. the snail slime's own molecules were actively involved in the network formation, and not only passive fractions of the solvent.

Table S3. Miscellaneous approaches for manufacturing of hydrogel bulks, based on nanoparticle-free snail slime solution: individually, the concentrations of cross-linker PEGDA (15 – 0 vol-%) and photoinitiator LAP (11.6 – 2.1 mg) were adjusted to a minimum level for generating stable three-dimensional networks.

	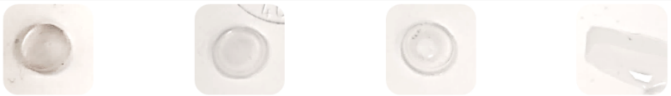			
**Crosslinker** - PEGDA (575 g mol^{-1})	**15 vol-%**	**10 vol-%**	**5 vol-%**	**0 vol-%**
**Photoinitiator** - LAP	**11. 6 mg**			
**Solvent** - Nanoparticle solution	**0.5 mL (1 mg mL^{-1})**			

	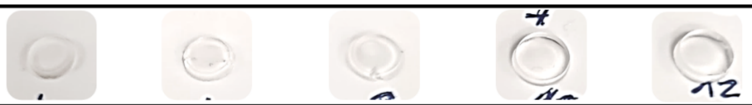				
**Crosslinker** - PEGDA (575 g mol^{-1})	**10 vol-%**				
**Photoinitiator** - LAP	**10.5 mg**	**8.4 mg**	**6.3 mg**	**4.2 mg**	**2.1 mg**
**Solvent** - Nanoparticle solution	**0.5 mL (1 mg mL^{-1})**				

2.3. Parameters of the POM-mould for photopolymerization

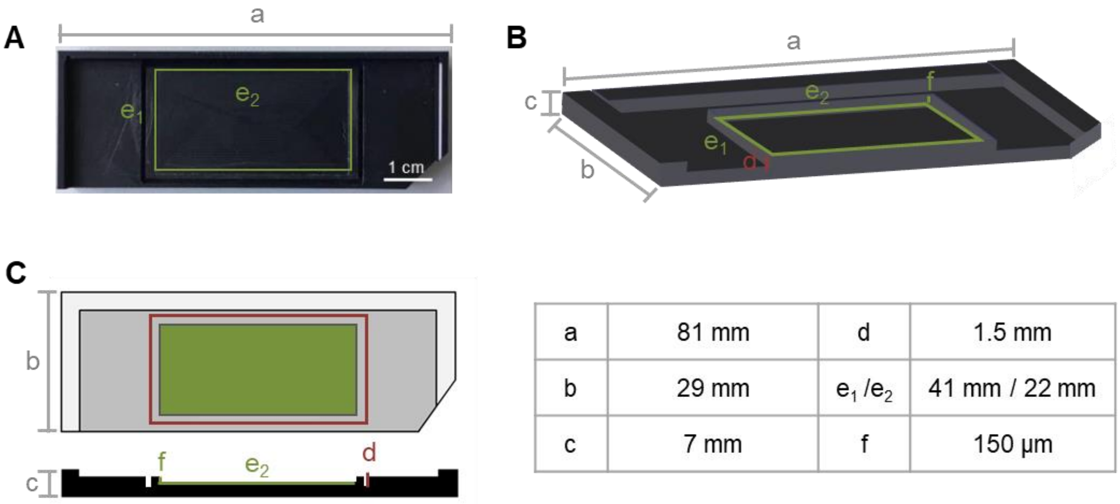

a	81 mm	d	1.5 mm
b	29 mm	e_1 /e_2	41 mm / 22 mm
c	7 mm	f	150 µm

Figure S5. Spatial structure and parameters of Poly(oxymethylene) (POM)-mould (a-f) for manufacturing of nanoparticle-comprising and nanoparticle-free snail slime-based hydrogel dots array by one-step photopolymerization for implementation into a microfluidic single-chamber reactor (**Figure S7A**): (A) photograph, (B) three-dimensional model, (C) top and side view.[5, 6]

3. Microfluidic experiments

3.1. Microfluidic setup and parameters

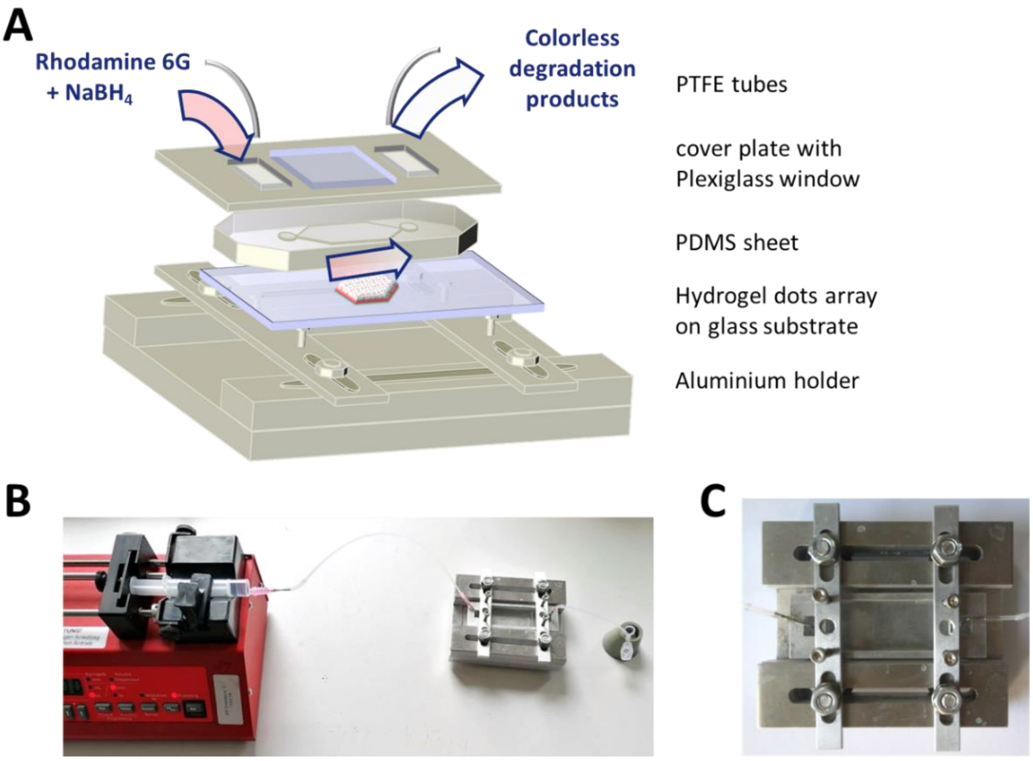

Figure S6. Microfluidic single chamber (A) Schematic illustration of the total assembly of the microfluidic chip from bottom to top: aluminum holder, snail slime-based hydrogel dots array bearing glass substrate, Poly(dimethylsiloxane) (PDMS) sheet containing the preformed reaction chamber, cover plate with a plexiglass window and Poly(tetrafluoroethylene) (PTFE) tubes and cannulas for supply and discharge of reaction solutions, comprising of 0.01 mM Rhodamine 6G and 0.5 mM $NaBH_4$ in MilliQ water; (B) Experimental setup of microfluidic experiments, including syringe pump for flow control (left) and Eppendorf tube (right) collecting the reaction solution after passing the microfluidic reactor;[4, 5, 7] the procedures for performing microfluidic experiments have been described as part of the main and experimental section.

A

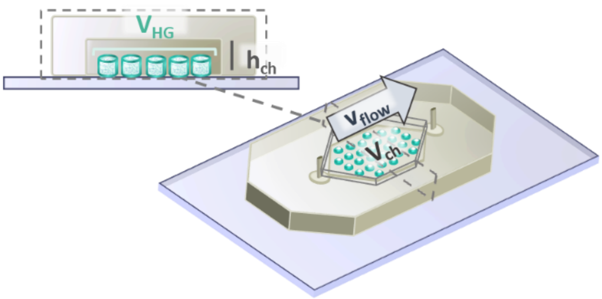

Residence time: $t_R = {}^{V_{Rct}}/_{v_{flow}}$

$$V_{Rct} = V_{ch} - V_{HG} = (A_{HG-Ar} \cdot h_{ch}) - V_{HG}$$

B

Design	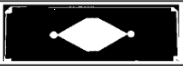
Number of chambers	1
Radius of hydrogel dots r_{dot} [µm]	175
Height of hydrogel dots h_{dot} [µm]	150
Number of hydrogel dots	227
Area of hydrogel array $A_{HG\text{-}Ar}$ [mm^2]	168
Volume of hydrogel per dot V_{dot}/ per chamber V_{HG} [µL]	0.01 / 3.3
Height of the chamber h_{ch} [µm]	160
Residence time t_R [s] (flow rate = 5 µL min^{-1}) (flow rate = 10 µL min^{-1})	283.0<br>139.7

Figure S7. Dimensioning and properties of the microfluidic, PDMS-based single chamber reactor, enclosing the snail slime-based hydrogel dots: (A) schematic illustration, including the parameters, and (B) calculation of the residence time, regarding the reaction solution of 0.01 mM Rhodamine 6G and 0.5 mM $NaBH_4$ (depending on the flow rate of 5 µL min^{-1} and 10 µL min^{-1}).[4, 6]

3.2. Nanoparticle-comprising hydrogel dots within a microfluidic single chamber reactor

Following the optimisation procedure for identification of the optimal snail slime concentration and determining the most promising hydrogel precursor composition, the implementation into microfluidic single chamber reactor under fluid flow-conditions for degradation of Rhodamine 6G was enabled. First, as introduced in **Figure 2**, three different concentrations of snail slime, were applied for achieving the final concentrations 0.5/2.5/5.0 mg mL^{-1} in nanoparticle solution. As expected, the results coincide with previously recorded fluorescence measurements: 0.5 mg mL^{-1} snail slime presented the highest decrease in intensity and consequently the highest degradation of Rhodamine 6G (**Figure S8B**). In addition, fluorescence spectra were captured for the collected samples of this series, i.e. at fixed interval (**Figure S8A**). Thereby, it is clearly recognizable that the largest drop occurred within the first 10 min before the fluorescence values remained constant for almost two hours.

Finally, the capability for the formation of NPs and embedding in hydrogel networks, was examined for four different slime species: *H.aspersa* and *H. pomatia*, Mucin and industrially extracted snail slime (**Figure S9**). Initially, all four samples were characterized by promising catalytic activity, showing continuous decrease and stagnation at 20 min period and fluorescence value between 7.7% (*H. pomatia*) and 4.6% (Mucin). Subsequently, the gold NPs were smoothly encapsulated in hydrogel dots arrays and examined under microfluidic

conditions (**Figure S9C, Figure 4B**). Thus, Mucin was confirmed as the most catalytically active slime species under fluid-flow conditions, while slime of *H.aspersa* yielded the weakest NPs in the long term. However, it should be noted that, the measurement results of all species are overall very close to each other.

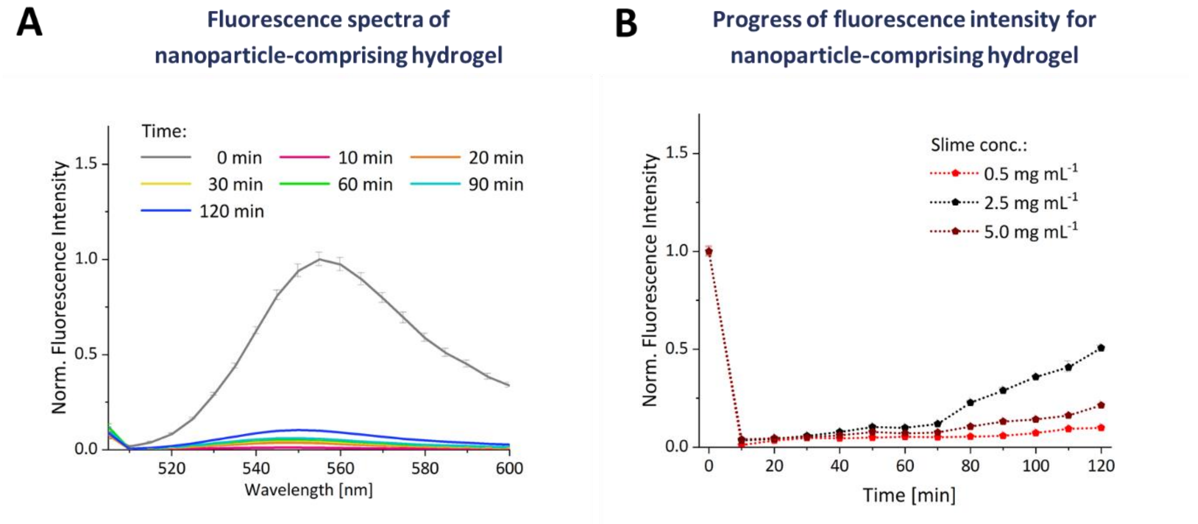

Figure S8. Fluorescence intensity measurements of gold nanoparticle-comprising, snail-slime based hydrogel dots arrays, derived from multiple snail slime concentrations: (A) Fluorescence spectra of different times over the total period of 120 min for 0.5 mg mL^{-1} snail slime solution, introduced in **Figure 2**, measured over an interval from 505 to 600 nm in 5 nm-steps, fluorescence intensity values were normalized to the maximum of the first measurement series (0 min); (B) Fluorescence development over the total period of 120 min for adapted snail slime concentrations of 0.5, 2.5 and 5.0 mg mL^{-1} from **Figure 2**, fluorescence intensity values were normalized to the respective start values; specimens of the reaction solution, initial comprising 0.01 mM Rhodamine 6G and 0.5 mM $NaBH_4$, were collected at the outlet of the microfluidic chip in 10 min-steps, measured diluted by 30 µL sample to 90 µL MilliQ water as triplet at an excitation wavelength of 478 nm and an emission wavelength of 556 nm.

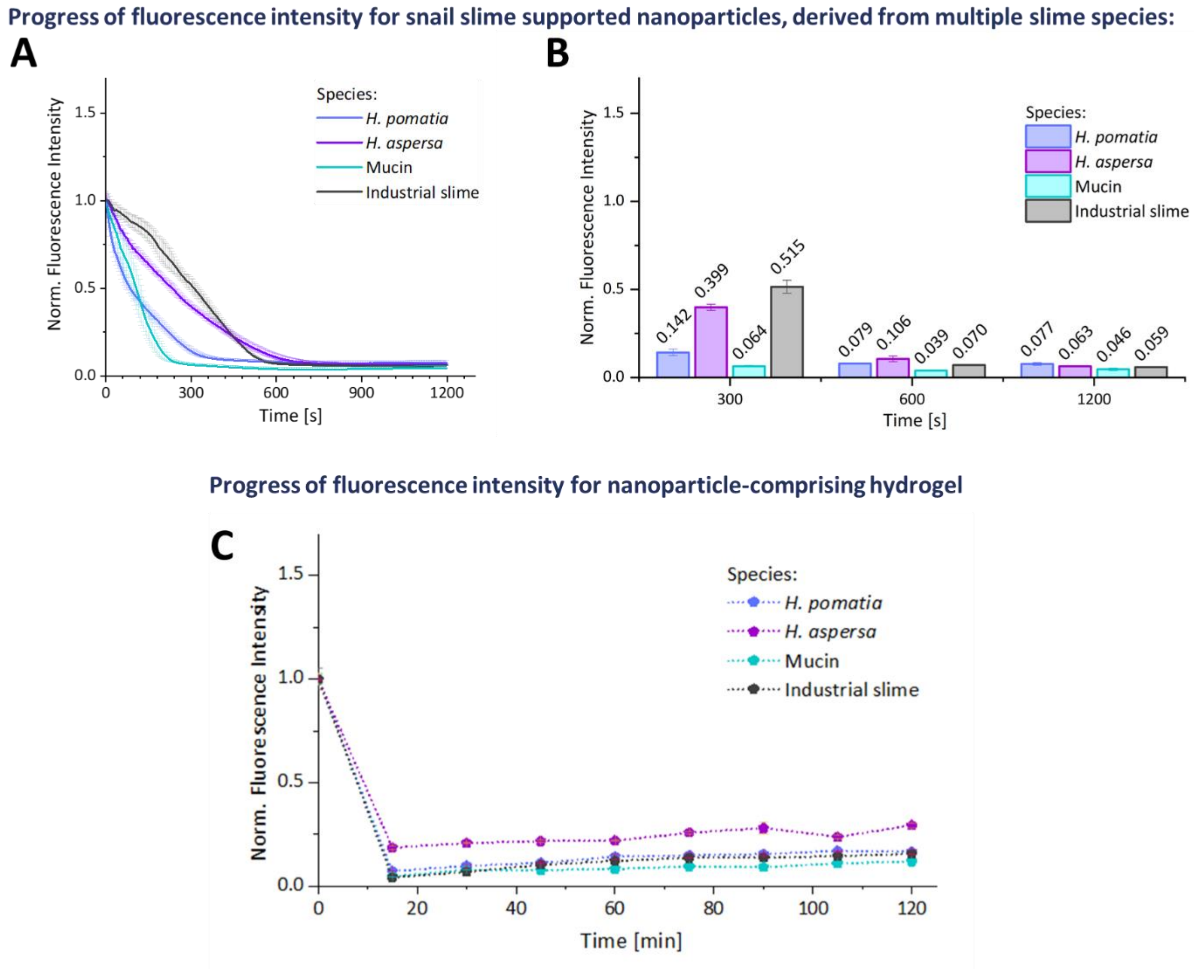

Figure S9. Fluorescence intensity studies for comparing the efficiency of different slime species, manually harvested slime from *H.aspersa* and *H. pomatia*, Mucin and industrially extracted snail slime, for the manufacturing of snail slime-supported gold NPs and physically entrapment into snail slime-based hydrogel dots arrays: (A) Fluorescence development by slime-supported NPs under stationary conditions within a microplate well over a total period of 20 min, normalized to the respective start value and (B) at specific times 5/10/20 min; therefor purified snail slime nanoparticle solution were mixed as 20 µL to a solution of 10 µL 10 mM $NaBH_4$ and 20 µL 0.1 mM Rhodamine 6G in 150 µL MilliQ water and monitored over 20 min at an excitation wavelength of 478 nm and an emission wavelength of 556 nm; (C) Implementation into a microfluidic single chamber reactor, as presented in **Figure 4C** (**Figure S6**), flushed with 0.01 mM Rhodamine 6G and 0.5 mM $NaBH_4$ at a flow rate of 10 µL min^{-1} for 120 min; samples were collected at the outlet of the microfluidic chip, measured diluted by 30 µL sample to 90 µL MilliQ water as a triplet at an excitation wavelength of 478 nm and an emission wavelength of 556 nm and finally normalized to the respective start values (0 min).

1. Rajoriya, S., S. Bargole, and V.K. Saharan, *Degradation of a cationic dye (Rhodamine 6G) using hydrodynamic cavitation coupled with other oxidative agents: Reaction mechanism and pathway.* Ultrasonics Sonochemistry, 2017. **34**: p. 183-194.
2. Rasheed, T., et al., *Reaction Mechanism and Degradation Pathway of Rhodamine 6G by Photocatalytic Treatment.* Water, Air, & Soil Pollution, 2017. **228**(8).
3. Ramakrishna, M., et al., *Green synthesis of gold nanoparticles using marine algae and evaluation of their catalytic activity.* Journal of Nanostructure in Chemistry, 2015. **6**(1): p. 1-13.
4. Koball, A., et al., *Boosting Microfluidic Enzymatic Cascade Reactions with pH-Responsive Polymersomes by Spatio–Chemical Activity Control.* Small Methods, 2024. **8**(12): p. 2400282.
5. Obst, F., et al., *One-step photostructuring of multiple hydrogel arrays for compartmentalized enzyme reactions in microfluidic devices.* Reaction Chemistry & Engineering, 2019. **4**(12): p. 2141-2155.
6. Jiao, C., et al., *Reversible Protein Capture and Release by Redox-Responsive Hydrogel in Microfluidics.* Polymers (Basel), 2022. **14**(2).
7. Simon, D., et al., *Hydrogel/enzyme dots as adaptable tool for non-compartmentalized multi-enzymatic reactions in microfluidic devices.* Reaction Chemistry & Engineering, 2019. **4**(1): p. 67-77.
